# Epidemic spread of *Pandoraea pulmonicola* in a cystic fibrosis center

**DOI:** 10.1186/s12879-015-1327-8

**Published:** 2015-12-26

**Authors:** Nicolas Degand, Romain Lotte, Célia Decondé Le Butor, Christine Segonds, Michelle Thouverez, Agnès Ferroni, Christine Vallier, Laurent Mély, Jacqueline Carrère

**Affiliations:** Laboratoire de Bactériologie, Centre Hospitalier Universitaire de Nice, Hôpital Archet II, Nice, France; INSERM 1065-Centre Méditerranéen de Médecine moléculaire, « Toxines microbiennes dans la relation hôte-agents pathogènes », Bâtiment Universitaire Archimed, Nice, France; Faculté de Médicine, Université de Nice Sophia-Antipolis, Nice, France; Observatoire Burkholderia cepacia, Laboratoire de Bactériologie, Hôpital Purpan, Toulouse, France; Laboratoire de Bactériologie, Hôpital Jean Minjoz, Besançon, France; Laboratoire de Bactériologie, Hôpital Necker-Enfants Malades, Paris, France; CRCM, Hôpital Renée Sabran, Giens, France; Laboratoire de Biologie, Hôpital Renée Sabran, Giens, France

**Keywords:** *Pandoraea pulmonicola*, Epidemic, Cystic fibrosis

## Abstract

**Background:**

*Pandoraea spp.* are recently discovered bacteria, mainly recovered from cystic fibrosis (CF) patients, but their epidemiology and clinical significance are not well known. We describe an epidemic spread of *Pandoraea pulmonicola* from 2009 in our CF center, involving 6 out of 243 CF patients.

**Methods:**

Bacterial identification used amplified ribosomal DNA restriction analysis (ARDRA), MALDI-TOF mass spectrometry (MALDI-TOF MS) and 16S rDNA gene sequencing. The clonal link between strains was assessed with pulsed field gel electrophoresis (PFGE) using XbaI. Clinical data were gathered for all patients.

**Results:**

The index case was chronically colonized since 2000. The main hypothesis for this bacterial spread was a droplet cross-transmission, due to preventive measures not being strictly followed. Antibiotic susceptibility testing revealed resistance to beta-lactams, ciprofloxacin and colistin. However, there was susceptibility to trimethoprim-sulfamethoxazole. All patients were chronically colonized with *Pseudomonas aeruginosa,* and the acquisition of *P. pulmonicola* resulted in chronic colonization in all patients. Three patients died, and two patients remained clinically stable, whereas one patient had a decline in lung function.

**Conclusions:**

This study, which is the first to describe an epidemic spread of *P. pulmonicola*, notes the potential transmissibility of this bacterial species and the need for infection control measures.

## Background

*Pandoraea* spp. are non-fermentative aerobic Gram negative bacilli first described in 2000 by Coenye et al. [1]. Initially, the genus *Pandoraea* was comprised of five bacterial species (*Pandoraea apista, Pandoraea pulmonicola*, *Pandoraea pnomenusa*, *Pandoraea sputorum* and *Pandoraea norimbergensis*). Since then, four other unnamed genomospecies and four other named species (*Pandoraea thiooxidans, Pandoraea oxalativorans, Pandoraea faecigallinarum and Pandoraea vervacti*) have been characterized [2–4]. These bacteria are ubiquitous in the environment and have been recovered from the soil, sea, and drinking water [5, 6]. In humans, *Pandoraea* spp. are mainly isolated from Cystic Fibrosis (CF) patients, and may cause chronic lung colonization [7–9]. Several *Pandoraea* species have been reported in CF patients. *P. apista* was involved in an epidemic spread in six CF patients in Denmark in 2003 [10]. More recently, *P. sputorum* was an agent of chronic CF lung colonization in Spain [8]. In addition to lung colonization, invasive infections due to *Pandoraea* species were also reported, although mostly in non-CF patients [11, 12]. However, a case of bacteraemia due to *Pandoraea* sp*.* was described in a CF patient [13]. This study reports an epidemic spread of *P. pulmonicola* in six patients attending our CF center and is the first description of an epidemic involving this bacterial species.

## Methods

### Patients

In 2008, *P. pulmonicola* was identified as a non-fermentative Gram negative organism, responsible for chronic lung colonization that started in 2000 in one of our CF patients. During 2009, five of the 243 patients attending our CF center contracted *P. pulmonicola*. In order to analyze the clinical impact of *P. pulmonicola* in the 6 patients, the following data were collected: age, sex, type of CFTR mutations, date of first *P. pulmonicola* isolation, associated pathogens, best forced expiratory volume in one second (FEV1) value per year, number of intravenous antibiotic courses per year, clinical status, transplantation and clinical outcome.

### Microbiology

Microbiological analysis of sputa was performed by growing sputa aerobically on both usual media and *Burkolderia cepacia* selective medium (BCSA) (Biomérieux, Marcy l’Etoile, France). Bacteria that grew on the BCSA were used for bacterial identification and antimicrobial susceptibility testing (AST). Bacterial identification for each suspected strain was performed using API 20NE strips (Biomérieux, Marcy l’Etoile, France), molecular techniques including amplified ribosomal DNA restriction analysis (ARDRA), *16S rDNA* gene sequencing, and MALDI-TOF mass spectrometry (MALDI-TOF MS). ARDRA was completed as previously described [14] with five restriction enzymes (*Alu*I, *Cfo*I, *Dde*I, *Msp*I, and *Xmn*I). The *gyrB* gene was amplified for further restriction analysis, according to Coenye [15], using the *P. apista* LMG 16407 type strain as a positive control. Moreover, the *16S rDNA*-based species-specific PCRs were performed as described by Coenye [16]. Identification with the *16S rDNA* gene sequencing was performed as previously described [17]. Sequence alignment used the NCBI/BLAST (www.ncbi.nlm.nih.gov/blast) and BIBI (https://umr5558-bibiserv.univ-lyon1.fr/lebibi/lebibi.cgi) programs. Sample preparation for MALDI-TOF MS analysis was conducted using MicroFlex LT with the Biotyper v2.3 database (Bruker Daltonics). Briefly, colonies from overnight bacterial cultures were smeared onto the target plate (1 spot per strain), and 1 μL of α-cyano-hydroxycinnamic acid was added. For strain comparison, one strain from each patient was tested with pulsed-field gel electrophoresis (PFGE) using *Xba*I, as previously described [18]. Antibiotic susceptibility was determined on Mueller-Hinton agar plates (Bio-Rad, Marnes-la-Coquette, France) using both the disk diffusion method with Bio-Rad disks (Bio-Rad, Marnes-la-Coquette, France) and the Epsilometer test (E-test) (Biomérieux, Marcy l’Etoile, France) according to the guidelines of the “Comité d’antibiogramme de la société française de microbiologie” (CASFM) 2013 for *Burkholderia cepacia* [19].

## Results

**Fig. 1:**
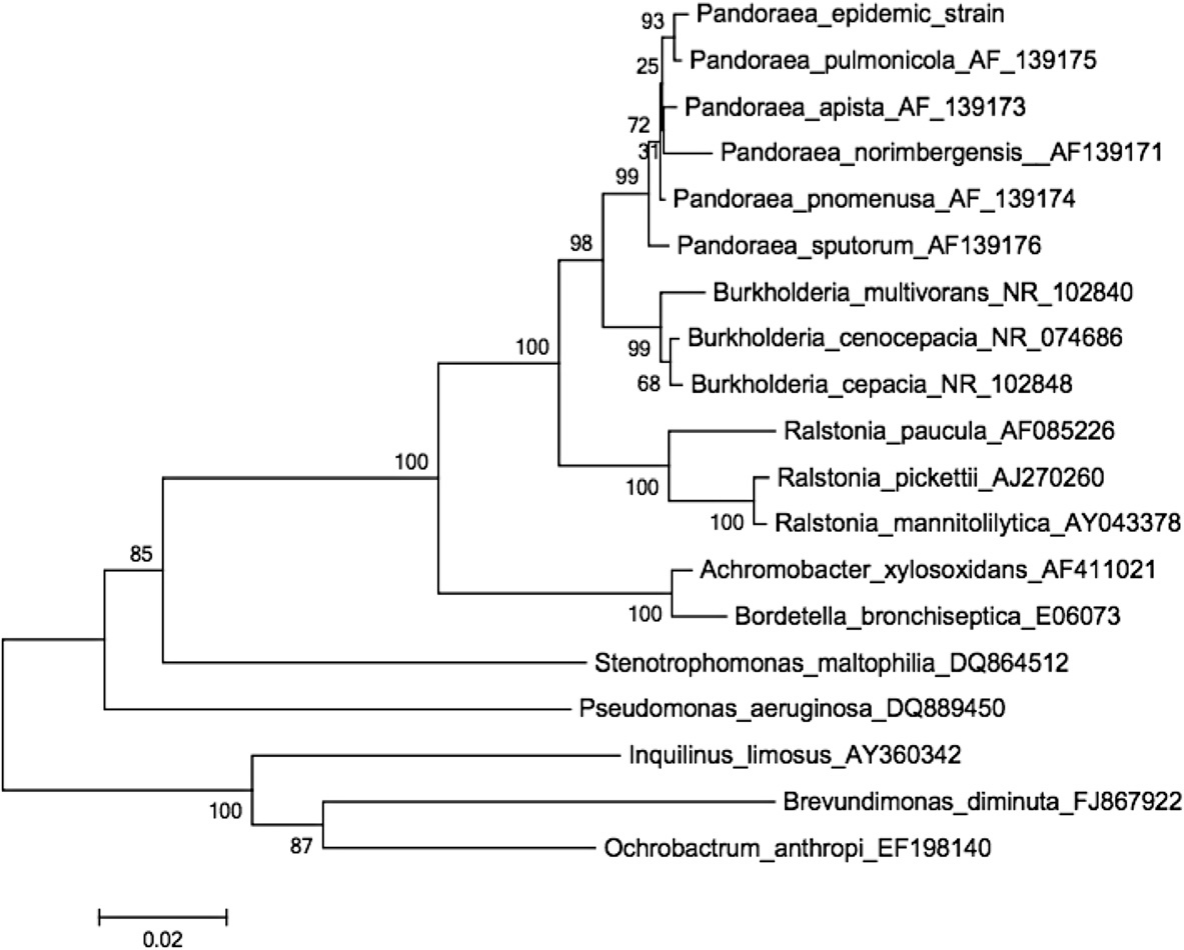
Phylogenetic tree based on the 16S rDNA sequence of our *Pandoraea* sp. epidemic strain. The tree was constructed using a neighbor-joining method, and 1000 bootstrap by using MEGA5 (Molecular Evolutionary Genetics Analysis version 5.0.) software as previously described (21). Values above the lines are bootstrap values expressed as percentages

**Fig. 2:**
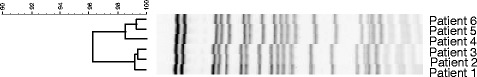
Dendrogram of the percent similarity between *Xba*I-digested genomic DNAs from *P. pulmonicola* isolates

The first identification of *Pandoraea pulmonicola* in our CF center occurred in January 2008 in a 64-year-old man (patient 1), who was previously considered chronically colonized with *Achromobacter xylosoxidans*, as detected by the API 20NE strip. This patient was also chronically colonized with mucoid *Pseudomonas aeruginosa*. The systematic recovery of this strain on the BCSA and its full resistance to colistin (absence of inhibition zone around a 50 μg disk) prompted the sending of one isolate to the Observatoire *Burkholderia cepacia* (Hôpital Purpan, Toulouse, France) for analysis. The strain was analyzed by ARDRA, which resulted in genus level identification of *Pandoraea* sp.; however, it could not discriminate between *P. apista* and *P. pulmonicola*. Among the *16S rDNA* based PCRs described by Coenye [16], a positive amplification was obtained with the primer pair appuF-panR, specific for *P. apista* and *P. pulmonicola*. However, the *gyrB* gene failed to be amplified. This strain was also analyzed by MALDI-TOF MS to confirm that the genus level identification of the *Pandoraea* sp. (log score value >2) matched the reference strain *Pandoraea* sp [2] 65 RLT. This identification was achieved through *16S rDNA* gene sequencing. Sequence alignment of the 655 bp amplicon using NCBI/BLAST (www.ncbi.nlm.nih.gov/blast) and BIBI (https://umr5558-bibiserv.univ-lyon1.fr/lebibi/lebibi.cgi) led to the identification of the strain as *P. pulmonicola* with 99.8 % sequence identity (654/655 bp) with *P. pulmonicola* strain CCUG 38759 (sequences NR_1151861 and AY268173 in NCBI and BIBI, respectively) and *P. pulmonicola* strain LMG 18106 (sequences NR_028750.1 and AF139175 in NCBI and BIBI, respectively, see the phylogenetic tree in Fig. 1). Retrospective identification of previous isolates from patient 1 recovered from 2000 to 2008 (50 sputum samples) rectified the identification as *P. pulmonicola* (misidentified as *A. xylosoxidans*) and was used to assess whether patient 1 was chronically colonized with *P. pulmonicola*. From March 2009 to December 2009, five additional patients acquired *P. pulmonicola*. Demographic, bacteriological and clinical data for the six patients are summarized in Table 1. Concerning bacterial identification, one strain from each patient was identified by ARDRA, *16S rDNA* gene sequencing and MALDI-TOF MS, which had the same results as patient 1. In order to analyze the clonality of the strains, one strain per patient was used for PFGE analysis. All strains displayed the same PFGE type, demonstrating a clonal link between strains (Fig. 2). Antimicrobial susceptibility testing was performed on one isolate per patient (recovered in 2009). All strains involved in the epidemic spread (patient 1 strains in 2009, and patient 2 to 6 strains) displayed the same antimicrobial susceptibility pattern with the same MIC values using the E-test methods. According to the interpretative standards for *Burkholderia cepacia*, the epidemic strain was resistant to piperacillin (>256 μg/mL), piperacillin-tazobactam (>256 μg/mL), ticarcillin-clavulanic acid (>256 μg/mL), ceftazidime (>256 μg/mL), cefepime (>256 μg/mL), imipenem (>32 μg/mL), meropenem (>32 μg/mL), ciprofloxacin (>32 μg/mL) and colistin (>256 μg/mL). The strain was susceptible to trimethoprim-sulfamethoxazole (0.5 μg/mL) and intermediate to rifampicin (8 μg/mL). MIC values for cefotaxime (3 μg/mL), ceftriaxone (8 μg/mL) and tigecyclin (4 μg/mL) could not be interpreted because no breakpoint values were available.

**Table 1:** Patients characteristics and clinical outcome

		Patient 1	Patient 2	Patient 3	Patient 4	Patient 5	Patient 6
Age (years)		64	23	39	29	18	18
Sex		M	M	M	F	M	M
CFTR mutation		1717-1GA/2789 + 5GA	F508del/F508del	F508del/F508del	F508del/F508del	F508del/R553X	F508del/F508del
Date of first P. pulmonicola isolation		2000	March 2009	June 2009	October 2009	November 2009	December 2009
Associated pathogens		mucoid *P. aeruginosa*	mucoid *P. aeruginosa*	mucoid *P. aeruginosa*	mucoid *P. aeruginosa*	*S. aureus*, mucoid. *P. aeruginosa, A. xylosoxidans, A. fumigatus*	*S. aureus*, mucoid *P. aeruginosa, A. fumigatus, G. argillacea*
Best Forced Expiratory Volume in 1 second (FEV1) value per year	2009	25	45	33	43	47	61
	2010	/	41	25	35	44	42
	2011	/	40	20	/	42	41
	2012	/	31	19	/	/	59
	2013	/	42	19	/	/	60
Number of intravenous antibiotic courses per year	2009	8	2	6	10	7	8
	2010	/	3	5	3	8	5
	2011	/	1	4	/	7	6
	2012	/	3	6	/	/	1
	2013	/	0	3	/	/	0
Organ transplantation		No	No	No (listed for heart and lung transplantation)	Lung transplantation (March 2010)	Lung and liver transplantation (April 2012)	Lung transplantation (January 2012)
Clinical outcome		Death (October 2009) from severe comorbidities	Stable	Decline in lung function	Death (April 2010) from post transplantation bacteremia due to P. aeruginosa	Death (May 2012) from severe multiple post transplantation organ failure	Stable

## Discussion

One of the patients attending our CF center was chronically colonized with *P. pulmonicola* since 2000, and five out of the 243 patients acquired this bacterium in 2009. Among *Pandoraea* species, *P. pulmonicola* was already reported in CF chronic lung colonization. This bacterial species was also reported to be the most predominant *Pandoraea* species among Irish CF patients [20]. Phenotypical characteristics of strains are consistent with the initial description made by Coenye et al. [1], since all strains displayed catalase and oxidase activity, polar motility and grew aerobically on usual media, such as Mueller-Hinton agar and also on BCSA. Notably, all strains recovered from patients 2 to 6 were non-pigmented, except for a brown pigmented strain recovered from patient 1 in 2008 and identified as *P. pulmonicola* by MALDI-TOF MS. The API20NE results were not reliable with an identity score of 58.9 % (T = 0.64) for the species *A. xylosoxidans* and an identity score of 22.2 % for *Alcaligenes faecalis* 2 (T = 0.6, biochemical profile: 0040476). Biochemical identification of *Pandoraea* spp. was not suitable because this genus is not included in the API20NE database. This explains the misidentification of the strain recovered from patient 1 prior to molecular identification. Previous studies have reported the misidentification of *P. sputorum* as *A. xylosoxidans* [11] and the misidentification of other *Pandoraea* species as *A. faecalis*, *A. denitrificans*, CDC IV C2 and *Acinetobacter* spp. [21]. ARDRA, as well as *16S rDNA*-based PCRs, resulted in a genus level identification of the *Pandoraea* sp., but could not discriminate between *P. apista* and *P. pulmonicola*. Interestingly, the *gyrB* gene restriction fragment length polymorphism (RFLP), which had been shown to be a reliable identification method for *Pandoraea* spp., could not be performed due to amplification failure of the *gyrB* gene for all the strains. Coenye et al. also reported low amplification of the *gyrB* gene from the three *P. pulmonicola* strains tested in their study [15]. Neither the *16S rDNA*-based specific PCR proposed by Coenye et al. in 2001 [16] nor the ARDRA test [14] were able to differentiate *P. pulmonicola* from *P. apista*. Nevertheless, our 655 bp *16S rDNA* gene sequence displayed more identity with the *P. pulmonicola* type strain LMG 18106 (99.8 %) than with the *P. apista* type strain LMG 16407 T (98.9 %). Comparing this sequence with other available *16S rDNA Pandoraea* strain sequences using the blast2N program (www.ncbi.nlm.nih.gov/blast/bl2seq/wblast2.cgi) resulted in 99.8 % homology with *P. pulmonicola* LMG 18106 T, 99.7 % with *P. pnomenusa* LMG 18087 T, 99 % with *P. apista* LMG 16407 T, 98.9 % with *P. sputorum* LMG 18819 T, and 98.4 % with *P. norimbergensis* LMG 18379 T. MALDI-TOF MS found the closest match with reference strain *Pandoraea* sp [2] 65 RLT (score value > 2 for all six strains). Interestingly, *Pandoraea* sp [2] 65 RLT displayed 99.8 % sequence identity with *Pandoraea pulmonicola* (accession number AF139175) and 99.5 % sequence identity with *Pandoraea pnomenusa* (accession number AY268170) (data provided by Bruker Daltonics). These results were consistent with the *16S rDNA* sequence identity obtained above. The results from ARDRA, *16S rDNA* sequencing and MALDI-TOF MS indicated that these methods should be used together in order to achieve an accurate identification at the species level.

In our assay, the epidemic strain was resistant to colistin, aminoglycosides, fosfomycin, pefloxacin, ciprofloxacin, ticarcillin, ticarcillin-clavulanic acid, meropenem, ceftazidime, and aztreonam, which was similar to previous reports for other *Pandoraea* species [8, 10, 11]. Until 2003, all *P. pulmonicola* strains recovered from patient 1 displayed a more susceptible phenotype with lower MIC values to piperacillin (32 μg/mL), piperacillin-tazobactam (24 μg/mL) and imipenem (3 μg/mL). This susceptibility pattern is similar to the *P. sputorum* strain described by Fernandez-Olmos et al. [8] with susceptibility to imipenem (MIC value of 4 μg/mL) and piperacillin-tazobactam (MIC value ≤ 16 μg/mL). Notably, patient 1 received piperacillin-tazobactam for the first time in 2007 and imipenem for the first time in January 2009, after the strain became resistant to these antibiotics. After 2003, all recovered strains from patient 1 displayed the same AST pattern as the epidemic strain (resistance to piperacillin, piperacillin-tazobactam and imipenem). Jørgensen et al. already reported intermediate susceptibility to ceftriaxone in *P. apista* strains [10]. Susceptibility to trimethoprim-sulfamethoxazole was also reported in *P. sputorum* strains [8].

Analyses of hospitalization periods for patients 1, 2, 3, 4, 5, and 6 are consistent with the chronological acquisition of *P. pulmonicola* (data not shown). In 2009, the source patient was hospitalized eight times. The clonality of the strains isolated from all six patients was assessed by PFGE analysis. The main hypothesis to explain the bacterial spread between patients is droplet cross-transmission. In our center, all patients have to follow standard precautions and are asked to use an alcohol hand rub. Specific droplet cross-transmission preventive measures consist in the use of respiratory masks for patients chronically colonized with *P. aeruginosa* and cohorting patients chronically colonized with the *B. cepacia* complex. In 2009, the number of patients in our center who were chronically colonized with *P. aeruginosa* was 150 out of 243 (61.7 %). The preventative measures were not strictly followed, and the patients had several contacts without respiratory masks, which is likely to have enabled droplet cross-transmission of *P. pulmonicola*. However, we cannot completely exclude an indirect droplet mediated cross-transmission or the possibility of exposure to a common bacterial source. Finally, by applying strict cross-transmission prevention measures, i.e. segregation of colonized patients in single hospitalization rooms with use of gowns for medical staff, consultations in specific rooms and on specific days, forbidden access to common areas, use of respiratory masks and use of specific spirometers with single use turbines and mouthpieces, the epidemic spread was controlled. In our study, all patients were chronically colonized with the mucoid *P. aeruginosa,* and associated pathogens were found in two patients. It would be interesting to determine whether chronic *P. aeruginosa* colonization is a condition that facilitates the acquisition and colonization of *P. pulmonicola*. All patients remained chronically colonized with *P. pulmonicola* after the date of first acquisition, which is consistent with previously described data regarding the ability of this bacterial species to chronically colonize lungs of CF patients. For example, in 2009, *P. pulmonicola* was recovered from 4/4 sputa (patient 2), 14/14 sputa (patient 3), 10/10 sputa (patient 4), 20/20 sputa (patient 5) and 6/6 sputa (patient 6). As for patient 1, the total number of positive sputa was 50 since the year 2000. The main features for all six patients concerning the clinical outcomes after *P. pulmonicola* acquisition are summarized in Table 1. Three patients died (patient 1, patient 4 and patient 5). Patient 1 died in October 2010 from severe comorbidities. Patient 4 died in the context of *P. aeruginosa* bacteraemia (*P. pulmonicola* was not retrieved in blood cultures). Patient 5 died in May 2012 from multiple-organ failure one month after lung and liver transplantation (blood cultures grew *P. pulmonicola*, *P. aeruginosa*, and *A. xylosoxidans*)*.* Several patients displayed declined lung function (Patient 3, Patient 4 and Patient 5). Two patients are clinically stable (patient 2 and patient 6). Patient 6 had a lung transplantation in January 2012 and is still chronically colonized with *P. pulmonicola* after bilateral lung transplantation. Altogether, the specific role played by *P. pulmonicola* in declined lung function is difficult to determine since it is associated with the chronic colonization of *P. aeruginosa*. However, recent data reported virulence of *P. pulmonicola* strains, mentioning that *P. pulmonicola* strains were more virulent than other *Pandoraea* species and could translocate through polarized lung epithelia with an in vivo virulence comparable to that of *B. cenocepacia* [21].

## Conclusions

Our study notes the role of *P. pulmonicola* as an emerging pathogen that can cause chronic lung colonization in CF patients. Identification tools need to be accurate and must be based on molecular techniques and MALDI-TOF MS. Cross-transmission preventive measures also need to be strictly followed to avoid an epidemic spread. Hopefully, the use of whole genome sequencing will provide significant additional information concerning the virulence of *P. pulmonicola*.

## Consent

Informed consent was obtained from the six patients that were reported in this article. For this retrospective observational study, ethics adherence was not required.

## Abbreviations

ARDRA: amplified ribosomal DNA restriction analysis
AST: antimicrobial susceptibility testing
BCSA: *Burkolderia cepacia* selective medium
CF: cystic fibrosis
FEV: forced expiratory volume
MALDI-TOF MS: matrix-assisted laser desorption/Ionization time-of-flight mass spectrometry
PFGE: pulsed-field gel electrophoresis
